# Introducing carbon quantum dot-Capivasertib drug carrier complex for enhanced treatment of breast cancer

**DOI:** 10.1371/journal.pone.0319206

**Published:** 2025-03-11

**Authors:** Moones Rahmandoust, Soroush Abdolrahimi

**Affiliations:** 1 Protein Research Center, Shahid Beheshti University, Tehran, Iran; 2 Department of Biochemistry and Bioprocess Engineering, Faculty of Life Sciences and Biotechnology, Shahid Beheshti University, Tehran, Iran; Fraunhofer USA, Inc. Center Midwest, UNITED STATES OF AMERICA

## Abstract

Capivasertib (AZD5363) is a 2023 FDA-approved pyrrolopyrimidine-derived compound that treats hormone receptor positive, HER2 negative metastatic breast cancer in adult patients. It is a novel pan-AKT kinase catalytic inhibitor in ER^ +^ breast cancer cell lines, including MCF7. The dominant influence of carbon quantum dots (CQDs) in combination with multiple chemotherapy drugs is also demonstrated as a drug delivery system that significantly enhances the effectiveness of cancerous tumour treatments by providing reduced side-effects, through targeted delivery of the drug, controlled release, enhanced solubility, permeability and retention. In this study, the impact of the conjugation of AZD5363 drug to N-doped, S-doped, and N/S-doped CQDs was investigated on inducing apoptosis by inhibiting the AKT signalling pathway in the MCF7 cell line. Initially, hydrothermal and pyrolysis methods were used to construct CQDs. Then, the synthesized quantum dots were conjugated with AZD5363 at three different concentrations, i.e., 0.03, 0.3, and 3nM. The MTT test results, on MCF7 cells, showed that although all the studied CQDs were biocompatible, the complex of N/S-doped CQD-AZD5363 at a concentration of 0.03nM was the most effective. After obtaining immunocytochemistry results, flow cytometry and cell invasion tests were employed to demonstrate the high potential of the introduced drug carrier complex in reducing AKT protein expression, induction of apoptosis and prevention of cell metastasis and invasion. According to these results, the binding of N/S-doped CQD to AZD5363 increases the effectiveness of this drug, with reducing the IC_50_ concentration, and more specificity to cancerous cells, introducing it as a suitable candidate for the treatment of breast cancer.

## Introduction

Breast cancer is a multifactorial process recognized as the most prevalent cancer among women. In recent years, the increase in the incidence of breast cancer has raised great concerns. Although chemotherapy is introduced as one of the most effective therapeutical approaches for the disease, its major limitation is the lack of tumour selectivity. Chemotherapy results in grievous side effects due to the diffusion into approximately all organs, damaging both cancerous and healthy cells [1]. Additionally, low drug loading efficiency and uncontrolled drug distribution limit the application of chemotherapy. Hence, finding new therapeutical approaches against this limitation is of great importance [2]. One approach toward selective targeting of the drug into the tumour is taking advantage of the abnormalities of the tumour area’s vascular architecture, including permeability factors hypervascularization, and extravasation within tumour tissues, as well as lack of lymphatic drainage [3].

Scientists have recently considered nanomaterials as effective delivery systems in cancer treatment to reduce the adverse effects of conventional therapies with specific targeted and sustained drug release capacity. Implementing novel drug delivery systems has proven promising by enhancing the therapeutic drug index and anti-cancer efficacy [4]. Carbon quantum dots (CQDs) are a new generation of oxygen-containing carbon nanomaterials which are less than 10 nm in size [5]. CQDs are among the famous candidates in medical applications due to their unique properties, namely their intense photoluminescence, small size, and biocompatibility, which can give them an excellent opportunity to be used as trackable drug carriers [6]. Recently, much progress has been made in the preparation of CQDs with modified chemical properties, to address therapeutic applications. One of the primary procedures for modifying CQDs is heteroatomic doping, which enhances their properties, including electrochemical and photoluminescence (PL) adsorption properties [7]. Doping effectively balances CQDs’ intrinsic properties by introducing atomic impurities (nitrogen, phosphorus, boron, etc.), among which, nitrogen and sulphur doping seem to have been the most promising heteroatoms. A study by Manioudakis et al. (2019) demonstrated that N-doping could regulate the carbon station characteristics of quantum dots, such as creating an electron charging station between neighbouring carbons and thus increasing the possibility of an added chemical [8]. Another study by Fan et al. (2017) showed that S-doping could increase the excitation state density and the polarization capacity of CQDs, which regulates the rotational density of CQDs [9]. The synergistic effects of multiple doping heteroatoms in carbon networks can immediately cause new densities of active sites for more effective drug loading in drug delivery systems [10]. Furthermore, doped CQDs show insignificant harm in healthy tissues, providing specificity to cancerous cells, owing to their diverse functional groups [11].

On the other hand, Capivasertib (AZD5363) is a pyrrolopyrimidine-derived compound that inhibits all three AKT isoforms (AKT1, AKT2 and AKT3) in enzyme assays and reduces the phosphorylation of AKT downstream protein substrates, such as GSK3β, PRAS40, P70S6K, and S6 [12,13]. There are studies showing the employment of the drug in various tumour models, including breast cancer, prostate cancer, leukaemia, and other advanced solid tumours [14]. However, the drug is recently FDA-approved a novel pan-AKT kinase catalytic inhibitor in ER^ +^ breast cancer cell lines. For example, Li et al. studied the impact of AZD5363 on breast cancer cell lines (MCF7, T47D, and ZR75.1). They showed a notable reduction of proliferation in all mentioned cell lines, due to the interference mechanisms of the AKT pathway by AZD5363 [15]. Another study examined the inhibition of AKT using AZD5363 in ER^ +^ breast cancer cell lines resistant to long-term oestrogen deprivation. Fox et al. proved that the combinations of AKT and IGF-IR/InsR inhibitors would be an effective treatment strategy against hormone-independent breast cancers. They showed that the treatment with AZD5363 reduced phosphorylation of the AKT/mammalian target of rapamycin (mTOR) substrates PRAS40, GSK3α/β and S6K, while inducing hyperphosphorylation of AKT at T308 and S473. Inhibition of AKT with AZD5363 suppressed growth and prevented the emergence of hormone-independent MCF7, ZR751 and MDA361 cells [16]. AZD5363 was also found to influence oestrogen receptor function in endocrine-resistant breast cancer and synergized with fulvestrant in vivo [17]. It decreased activated or phospho-AKT (p-AKT)/mTOR targets, leading to a reduction in ERα-mediated transcription in a context-specific manner and a concomitant decrease in the recruitment of ER and CREB-binding protein to oestrogen response elements located on the TFF1, PGR, and GREB1 promoters, showing a dose-dependent decrease in proliferation in MCF7, T47D, and ZR75.1 cell lines (GI50 <  500 nmol/L), where the T47D-LTED and ZR75-LTED were the most sensitive of the lines (GI50 ~  100 nmol/L) [17].

In line with the path of progress in clinical trials on AZD5363-treated breast cancer cells to FDA approval [18], Choi et al. assessed the effect of the drug on the phosphoinositide 3-kinase (PI3K)/AKT/mTOR pathway. They reported that alternative pathways are activated in AKT-inhibitors-treated cells for escaping the inhibition of the cell growth, i.e., the levels of phosphorylated glycogen synthase kinase 3 beta were reduced in the Hs578T cell line of breast cancer [14]. More recently in 2020, Gris-Oliver et al. provided an insight into the mechanisms of resistance to AZD5363 and its combination with paclitaxel in HER2-negative metastatic breast cancer, introducing the drug as a catalytic inhibitor with promising activity in combination with paclitaxel in triple negative metastatic breast cancer harbouring PI3K/AKT-pathway alterations and in oestrogen receptor-positive breast cancer in combination with fulvestrant [19]. They analysed genetic and proteomic markers in twenty-eight HER2-negative patient-derived xenografts (PDXs) and inpatient samples, and correlated to AZD5363 sensitivity as a single agent and in combination with paclitaxel. Four PDX were derived from patients receiving AZD5363 in the clinic which exhibited concordant treatment response. Mutations in PIK3CA/AKT1 and the absence of mTORC1-activating alterations in mTOR or TSC1, were associated with sensitivity to AZD5363 monotherapy. Moreover, resistant PDXs exhibited low baseline pAKT S473 and residual pS6 S235 upon treatment, suggesting that parallel pathways bypass AKT/S6K1 signalling in these models [19]. Furthermore, Robertson et al. demonstrated that treating breast cancer patients with AZD5363 reduced p-Akt/mTOR expression, leading to a decrease in oestrogen receptor-mediated transcription as the primary chemotherapy strategy for breast cancer [20]. Considering the significance of the AZD5363 drug and the extent of its influence on solid tumours on one hand, and the importance of the strategy of incorporating nanoparticle with chemotherapy drugs, on the other hand, this drug was selected to be incorporated with CQDs to investigate its influence on MCF7 cell line, for the first time. Nanoparticles in general and carbon-based nanomaterials, to be more precise, are nontoxic particles with proved capabilities in terms enhanced effectiveness in treatment of cancerous tumours by providing reduced side-effects, through targeted delivery of the drug, controlled release, enhanced solubility, permeability and retention, making nanoparticle-based drug delivery systems one of the intuitive approaches to increase its inhibitor’s activity and reduce its toxicity in healthy cells [21–25], The complex of CQD-AZD5363 was expected to significantly boost the drug features because, multiple studies have demonstrated that applying drugs in combination with CQDs induces highly significant effects. For example, in a study conducted by Zavareh et al. (2020), the fluorouracil-chitosan N-doped CQDs (NCQD)-aptamer-5-FU was used for targeted delivery of 5-FU in the treatment of breast cancer. The results illustrated that the designed system could effectively kill MCF7 cells, which could be used as a potential nanocarrier in treating breast cancer [26]. In another study, Cao et al. (2007) demonstrated that using mobile surface CQDs in multi-motion bioimaging to enter MCF7 cells in breast cancer had a significant potential to cross the cell membrane and hence cytoplasm labelling [27]. In another study, doxorubicin (DOX) was covalently attached to CQDs. It showed that CQDs-DOX were rapidly absorbed by A549 cancer cells, and fluorescence microscopy confirmed that CQDs-DOX are mainly localized in the cytoplasm. However, DOX was also detected in the cell nucleus [28]. Consistently, an investigation by Mehta et al. demonstrated that applying CQDs as a carrier of isoprenyl has a significant impact on increasing the permeability of Hela cancer cells to the desired drug [29]. In this regard, the study of Shu et al. also showed that the CQDs conjugation with curcumin increases the load of the drug into Hela cancer cells too [30].

Considering the efficacy of AZD5363 in the treatment of breast cancer by inhibiting the AKT signalling pathway and the impact of CQDs as a promising drug delivery system, in this study, the combination of AZD5363 with three types of CQDs including, N-doped, S-doped, and N/S-doped CQDs were introduced to investigate the cell death pathway by inhibiting AKT signalling on the MCF7 cell line.

## Methods and materials

### CQDs synthesis

Hydrothermal and pyrolysis techniques were used to synthesize N-doped, S-doped, and N/S-doped CQDs. The preparation methods for CQDs were optimized as follows:

### N-doped CQDs

Urea (0.20 g) (Merck, Germany) and ammonium hydrogen citrate (0.20 g) (Merck, Germany) were mixed homogeneously in an agate mortar and the dry powder was placed in a glass beaker to be heated at 180°C for 1 h in ambient air, for the synthesis of the NCQDs using pyrolysis method. Then, the obtained dark sediment, containing NCQDs, was diluted and neutralized to pH =  7 by 1M NaOH (Merck, Germany). The final solution was then centrifuged to separate the large particles for 30 min at 20K rpm and filtered [31].

### S-doped CQDs

In order to synthesise S-doped CQDs (SCQDs), two salts, namely 1.3 g of trisodium citrate (Merck, Germany) and 2.4 g of sodium thiosulfate (Merck, Germany) were added to 50mL of distilled water [32]. The resulting transparent solution was then transferred into a Teflon-lined reactor and was heated at 200°C for 6 h. After reaching room temperature, the final SCQD solution was centrifuged and filtered to remove large particles.

### N/S-doped CQDs

N/S-doped CQDs (NSCQDs) were prepared using citric acid monohydrate (2.00 g) (Merck, Germany) and of L-cysteine (1.0 g) (Merck, Germany), which were well dissolved in 5 mL of distilled water on a magnetic stirrer for 15 minutes. The resulting solution was then placed in an oven at 70°C for 12 h to evaporate, leading to a thick liquid, which was transferred to a Teflon-lined reactor to be hydrothermally heated at 200°C for 3 h. Next, the solution was left to reach room temperature for about 3 h without opening the reactor. Finally, the synthesis solution was diluted using 50 ml of distilled water, and it was placed on a magnetic stirrer for 20 minutes. The pH of the final solution was measured and set to pH =  7 by using NaOH (1M) and it was centrifuged and filtered to remove large particles [33].

### Characterization of CQDs

Different methods were used to determine the characteristics of NCQDs, SCQDs, and NSCQDs. Dynamic Light Scattering (DLS) was done by a Malvern Nano ZS (Malvern Instruments, Malvern, UK). All experiments were conducted with three replications, at room temperature, at the scattering angle of 90 degrees, using a 632.8 nm HeNe laser light source, with a maximum intensity of 10 mW. Bright-field high-resolution transmission electron microscopy (HRTEM) was used to analyse the morphology and particle sizes, together with the Easyscan 2 Nanosurf (Liestal, Switzerland) atomic force microscopy (AFM).

Investigation on the chemical structures of synthesized CQDs was performed using Fourier-transform infrared (FTIR) spectroscopy (Thermo-Nicolet NEXUS 470, Illinois, USA), and X-ray Photoelectron Spectroscopy (XPS) (PHI 5000 Versaprobe, Physical Electronics, Minnesota, USA).

The X-ray diffraction (XRD) spectrum was taken for 2*θ* between 1 to 80 degrees, using a Cu *Kα*1 X-ray radiation source (DMAX-2500 diffractometer, Rigaku, Japan). Finally, fluorescence spectroscopy was executed to investigate the optical performance of synthesized CQDs, using PerkinElmer’s LAMBDA 950 UV/Vis/NIR Spectrophotometer, USA.

### Synthesis of the complex

A solution of CQD-AZD5363 (AstraZeneca, UK) was made by combining AZD5363 at a concentration of (0.03, 0.3, and 3nM) in equal proportions to equal concentrations of the synthesized CQDs and placed on a magnetic stirrer in a dark environment for 48 h. The achieved suspensions were then dialysed at room temperature in DI water as the buffer, using 3.5K dialysis bag for 24 h, to remove the residual reagents.

Enzyme-linked immunosorbent assay (ELISA) reader (Infinite, M200 PRO, Tecan, USA) was used at 490 nm, specific to AZD5363 to determine the effective concentration of the loaded drug in the complex. The data was used to calculate the efficiency of conjugation process by obtaining the percentage of the drug concentration in the complex and comparing it with the initial drug concentration used.

### Cell culture

Human breast cancer cell line MCF7 was seeded in Dulbecco’s modified Eagle medium (DMEM, Gibco-Invitrogen, USA) supplemented with 10% fetal bovine serum (FBS) (Gibco-Invitrogen, USA), 1% penicillin along with streptomycin (Gibco-Invitrogen, USA), and 1% non-essential amino acids (Gibco-Invitrogen, USA) at 37°C in a humidified atmosphere with 5% carbon dioxide (CO2). The medium was changed every 2-3 days, and the cells were passaged until reaching 80% confluency using 0.05% trypsin-EDTA (Gibco-Invitrogen, USA) to detach the cells.

### MTT cell viability assay

MCF7 cells were used for cell viability assay via the MTT method. In summary, MCF7 cells were seeded at a density of 5000 cells/well in a 96-well plate and left overnight. On the day after, they were treated using increasing concentrations of NCQDs, SCQDs, NSCQDs, AZD5363-NCQDs, AZD5363-SCQDs, AZD5363- NSCQDs, and AZD5363 alone (0.03, 0.3, and 3nM) in DMEM and incubated for 72 h. Next, the medium was replenished with 20 μl of MTT solution (0.5 mg/ml in phosphate-buffered saline (PBS)) (Sigma, USA) and incubated at 37°C for 4 h. Then, the medium was removed, and 100 µl of DMSO to solubilize MTT-formazan, and the absorbance was evaluated by a microplate reader (Eon, BioTeck, USA) at 570 nm. MTT test was done in triplicate for each concentration, and half-maximal inhibitory concentration (IC_50_) values were used to explain drug concentrations that reduced absorbance to 50% of control values.

### Immunocytochemistry assessment

Immunocytochemical (ICC) staining was performed to investigate the inhibition of AKT signalling in MCF7 cells, after treatment with DMSO (as the solvent of formazan), PBS (as the solvent of AZD5363 and NSCQD), NSCQDs (0.3nM), and the NS-AZD complex (0.3nM). The MCF7 cells with a density of 1 × 105 cells/well were seeded in 6-well plates and administered with an IC_50_ dose of candidate CQDs for 72 h. After discarding the medium, 4% paraformaldehyde was used to fix the MCF7 cells for 20 min and then rinsed three times with PBS. Then, the cells were permeabilized with 0.3% Triton X-100 (Sigma-Aldrich, Germany) and incubated with 1% bovine serum albumin (BSA) (Sigma-Aldrich, Germany) for 1 h at room temperature, followed by incubation with anti-mouse or anti-rabbit horseradish peroxidase (HRP)-conjugated antibodies (1/100 diluted, Cell Signalling Technology).

At the next step, cells were washed with PBS and incubated with fluorescein-labelled secondary antibody (1/100 ratio) for 45 min in the dark at room temperature nuclei were counterstained with 4’,6- Diamidino-2-Phenylindole (DAPI solution, 0.5 µg/ml) (Thermofisher, USA) and the images of the stained cells were taken by fluorescent microscope with DAPI and fluorescein isothiocyanate (FITC) filters (Zeiss, Germany).

### Apoptosis assessment

Apoptosis-mediated MCF7 cell death was evaluated by applying Annexin V-FITC Apoptosis Detection Kit (BD Bioscience) based on the manufacturer’s instruction. Briefly, MCF7 cells at a density of 5000 cells/well were cultured in a 12-well plate treated with an IC_50_ dose of candidate CQDs for 72 h. Next, 0.05% trypsin-EDTA was used to detach the cells and resuspend them with 200 µl of binding buffer. In the following step, cells were incubated with propidium iodides (PI) (5 µl) and Annexin-V-FITC (5 µl) for 5 minutes at room temperature. Data acquisition and analysis were performed using a flow cytometer (CyFlow SL, Partec- Germany) with the software FlowJo Version 7.6.1 (Ashland, OR). V-FITC and PI-stained cells were positive for cells at all stages of apoptosis and dead and late apoptotic cells, respectively.

### Detection of the cell migratory capacity

Cell migratory capacity was studied using the scratch-wound assay. The MCF7 cells were cultured at 1 × 10^6^ cell/well in 6‐well culture plates till reaching approximately full confluency. The scratch‐wounds were performed using a cell scraper (Nunc, Inc., Naperville, IL, USA). Next, an IC_50_ dose of the candidate CQD was added, and the cells’ movement was photographed at regular intervals (0 and 72 h).

The inverted microscopy was used to obtain the images of cell migration and assessed using an image processing software ImageJ (National Institute of Health, Bethesda, MD, USA).

### Statistical analysis

The numerical data obtained by ImageJ software was statistically analysed with Prism software version 9 (GraphPad Software, USA). One-way ANOVA test and Tukey method were performed to compare the data from the different experimental groups. Data were expressed as mean ±  standard deviation with a significance level of p-value ≤ 0.05.

## Results

### Characterization of CQDs

**Fig 1:**
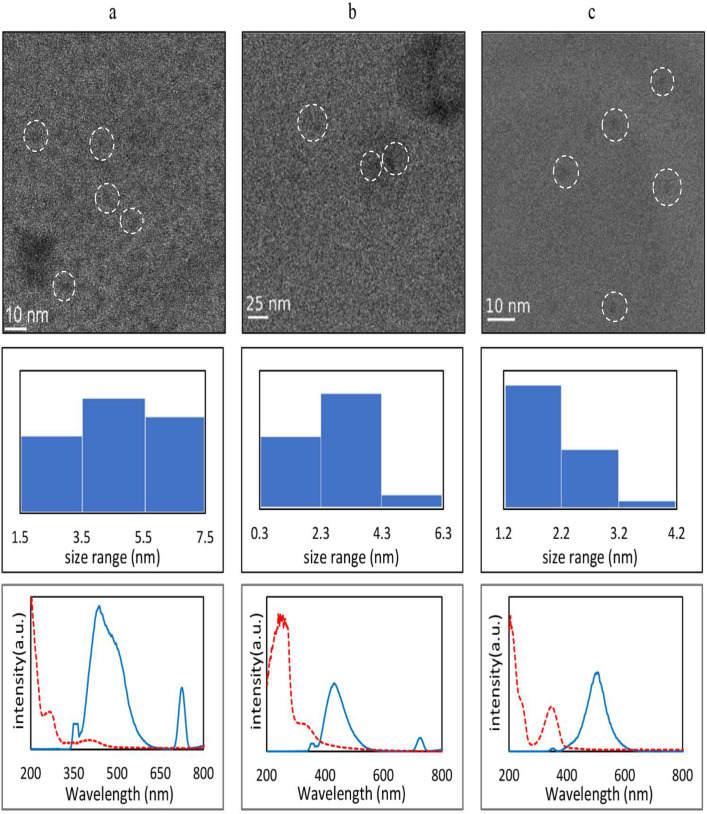
From top to down: HRTEM, histogram and the PL absorption/emission (λ_ex_ = 360 nm) of (a) NCQDs, (b) SCQDs, and (c) NSCQDs.

As shown in Figs 1a through 1c, according to the HRTEM and DLS results, the average sizes of NCQDs, SCQDs, and NSCQDs were detected to be about 4.9 ± 0.3 nm, 2.8 ± 1.3 nm, and 2.1 ± 0.7 nm, respectively, with obtained zeta potentials in three replications equal to -0.6 ± 0.3 mV, -2.6 ± 1.1 mV, and -0.3 ± 0.1 mV, respectively. The PL investigation was done to confirm the formation of the fluorescent CQDs. NCQDs had two major absorption wavelengths of about 270 nm and 370 m. When excited with 360 nm UV radiation, the NCQDs were emitting two wavelengths of about 440 nm and 730 nm, with a visible greenish colour. SCQDs had a maximum visible blue-emission wavelength at about 420 nm and a tiny peak at about 730 nm, when excited by 360 nm UV wavelength. The UV-excited NSCQDs, on the other hand, had visible radiation with a wavelength of nearly 520 nm (Figs 1a through 1c).

**Fig 2:**
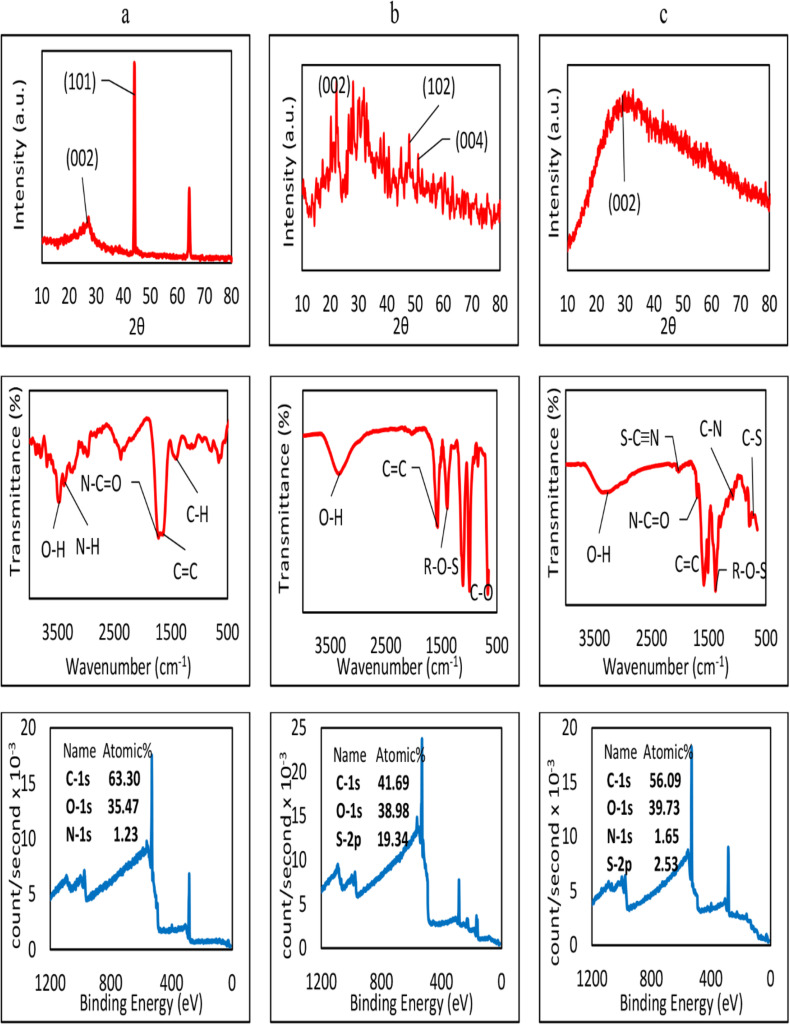
From top to down: the XRD, FTIR and the full XPS spectrum of (a) NCQDs, (b) SCQDs, and (c) NSCQDs.

Figs 2a through 2c illustrate the XRD, FTIR and the full XPS spectrum of the CQDs. As can be observed, the characteristic (001) XRD peak is observed below 2θ =  20 degrees. In the range between 20 and 30 degrees, (002) peaks can be seen. Between 40 and 50 degrees, (100), (101) and (102) graphitic carbon crystalline phases are remarkable. The (004) is placed between 50 and 55 degrees, whereas (110) is seen in the range of 75-80 degrees [34]. As it can be seen, regardless of the synthesis technique or the sources used as precursors, a broad XRD peak is sometimes observed at 2θ between 20 to 40 degrees, which does not belong to graphite (i.e., around 25˚), showing that the crystal domains are not limited to graphite carbon, but the structure contains some other amorphous cross-linked polymer skeletons in these CQDs [35].

The elemental composition of CQDs was obtained using XPS and FTIR analysis. The FTIR spectrum confirms that in NCQDs, shown in Fig 2a, the N-C = O carbonyl stretching vibration of amide and the N-H amine stretching, in the ranges of 1800-1600 cm^-1^ and 3400-3300 cm^-1^, respectively, are observed. In SCQDs, The O-S bond of organic sulphate is significant between 1370 and 1420 cm^-1^ and C-O bending 1150-1085 cm^-1^ (Fig 2b). In NSCQDs (Fig 2c), the N-C = O and S-C ≡ N, carbonyl amide stretching vibrations, are observed in the ranges of 1800-1600 cm^-1^ and 2175-2140 cm^-1^, respectively. The C-N and C-S bonds can be distinguished at 1072, and 717 cm^-1^, respectively [33]. A sharp peak of the organic sulphate O-S bond is observed at 1379 cm^-1^ [36].

**Fig 3:**
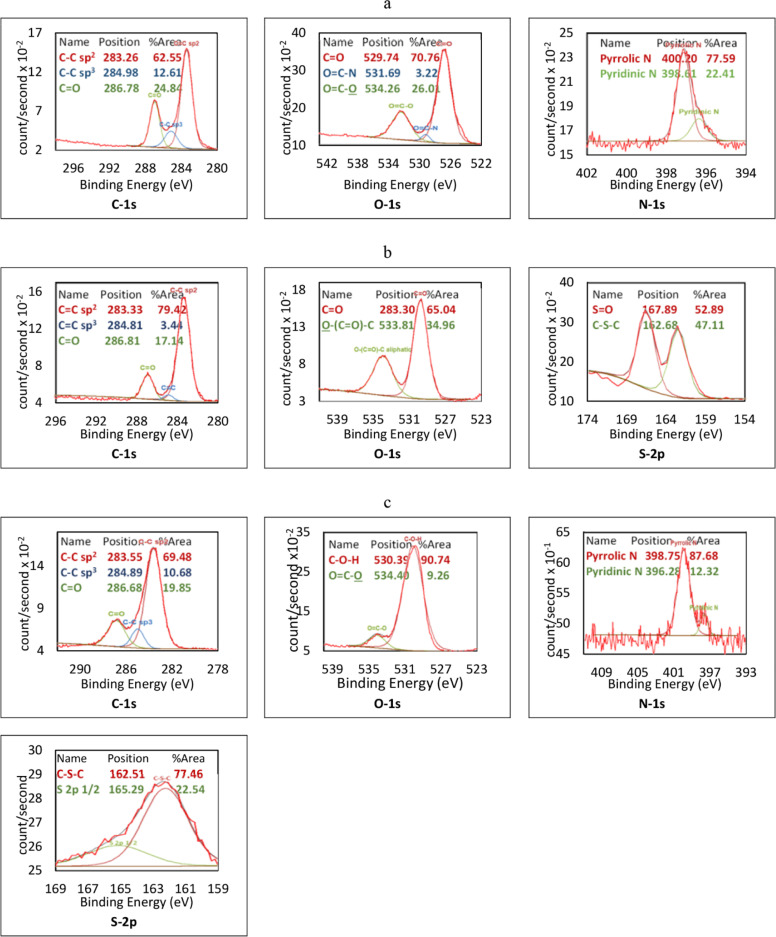
The high-resolution XPS spectrum of (a) NCQDs, (b) SCQDs, and (c) NSCQDs.

In the XPS survey of NCQDs, as shown in Figs 2a and 3a, the peaks C1s, O1s, and N1s, each show 63.3%, 35.5% and 1.2% of the atomic content of the NCQDs, respectively. The high-resolution C1s spectrum shows three peaks at 286.78 eV, 284.98 eV and 283.26 eV, belonging to C = O, C-C sp^3^ and C = C sp^2^, respectively [37], which is in agreement with FTIR results. Two types of pyridinic and pyrrolic C-N bond configurations are observed based on O1s and N1s spectra, as in the N-C = O bond at 531.69 [38]. In the case of SCQDs, the full XPS spectrum in Figs 2b and 3b reveal the existence of a rather high sulphur content of about 19.3%. The C1s spectrum shows that the C = C sp^2^ bond at 283.55 eV is the most dominant, covering 79.4% of the spectrum, as the FTIR diagram also confirms. The S = O bond at the binding energy of 168.89 eV, and the C- S-C bond at 162.68 eV, are the major S bonds as the high-resolution S 2p XPS results.

In the NSCQD’s full and high-resolution XPS spectrum in Figs 2c and 3c ~ 56.1% of carbon, ~ 39.7% of oxygen, ~ 1.7% of nitrogen and ~ 2.5% of sulphur bonds are reported. In the C1s spectrum, the sp^3^ and sp^2^ carbon-carbon bonds are seen at 284.89 and 283. 55 eV, respectively, along with C = O at 286.68 eV. The N1s spectrum shows the pyridinic and pyrrolic C-N bonds at396.28 eV and 398.75 eV binding energies, whereas C-S-C and S2p ½ are observed at 162.51 eV and 165.29 eV peaks of the S2p spectrum, respectively. The bonds observed in the NSCQDs’ structure originate from the precursor L-cysteine and citric acid, as admitted by FTIR and XPS results, which lead to their excellent optical properties and good water solubility [39].

### Characterization of the complexes

**Fig 4:**
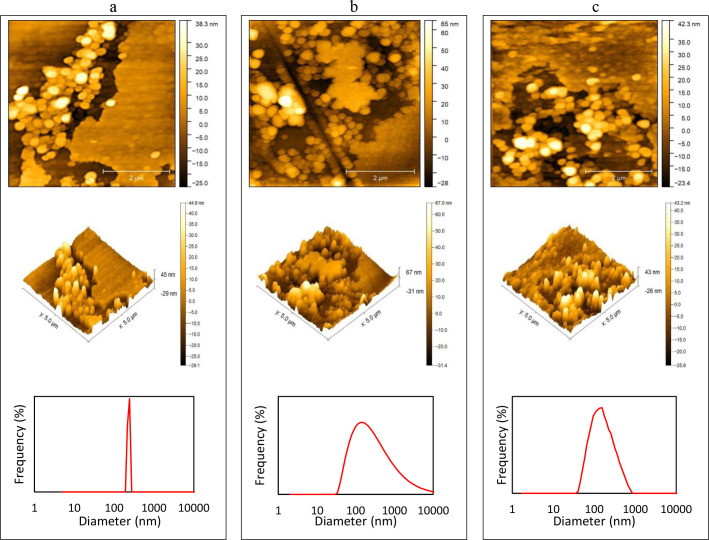
AFM and DLS results obtained for (a) NCQD-AZD5363, (b) SCQD-AZD5363, and (c) NSCQD-AZD5363 complexes.

After synthesizing NCQDs, SCQDs, and NSCQDs, and conjugating them to AZD5363 to form the targeted complexes, their characteristics and therapeutical impacts were investigated. At first, the effective sizes of the complexes were studied, using DLS and AFM. The results show average diameters of 243.5 ± 23.2 nm (Fig 4a), 145.8 ± 74.4 (Fig 4b), and 175.1 ± 43.3 (Fig 4c) for the synthesized NCQD-AZD5363, SCQD-AZD5363, NSCQD-AZD5363 complexes, respectively. According to these results, from the newly developed drug carrier systems, S- and N/S-doped CQDs are suitable in terms of size, to enhance the permeability and retention of the drug, as the scientific concept of enhanced permeability and retention effect (EPR) suggests. Based on this concept enlarging the size of the drug carrier to an optimum range between about 100 and 200 nm facilitates the penetration of the drug carrier to underdeveloped, leaky endothelium tumour blood capillaries and their accumulation in tumour tissues for longer periods, due to the lack of lymphatic drainage [3].

### Cells viability assessment

**Fig 5:**
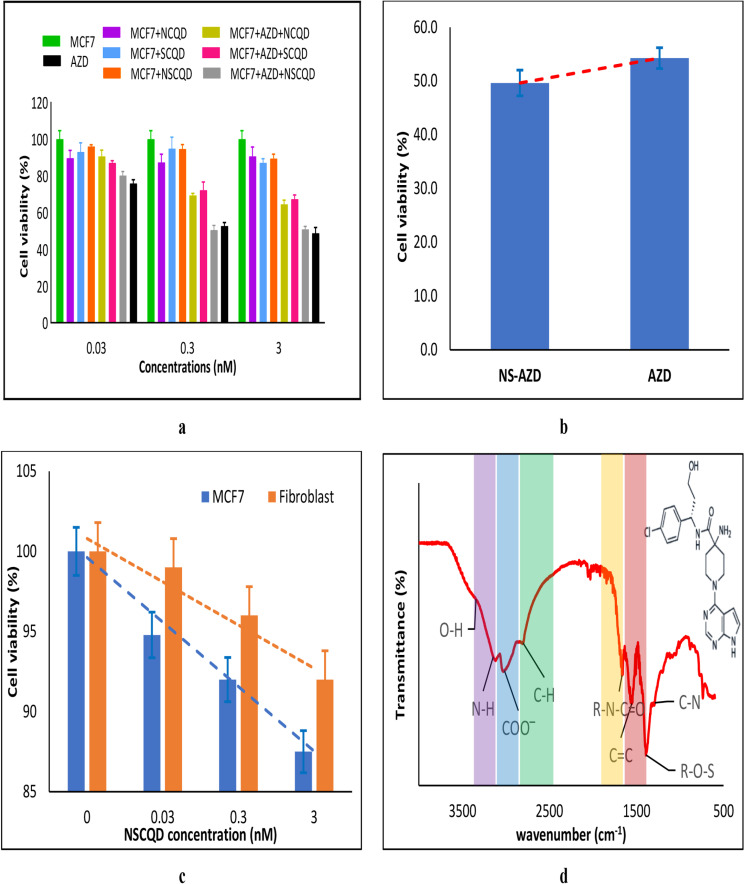
MTT test results on MCF7 for (a) NCQDs, SCQDs and NSCQDs, alone and when conjugated with AZD5363 after 72h at three concentrations of 0.03, 0.3 and 3nM, (b) when treated by 0.3nM NS-AZD complex, compared to the drug alone, and (c) after treatment on Fibroblast and MCF7 cells after 24h, at three concentrations of 0.03, 0.3 and 3nM; (all standard deviations are calculated after three replications) and (c) FTIR of NS-AZD (inset: AZD5363 chemical structure).

An MTT assessment was done on the MCF7 cell line, treated with the synthesized CQDs and AZD5363-CQD complexes at three different concentrations, i.e., 0.03, 0.3, and 3nM, as shown in Fig 5a. Based on the results, the percentage of cell viability in the groups of MCF7 cells treated with the CQDs, in all tested concentrations of 0.03, 0.3, and 3nM, are almost similarly insignificant after 72 h. Considering the IC_50_ value, the rate of viability of MCF7 cells has decreased in the NSCQDs-AZD5363 (NS-AZD)-treated group, compared to the group treated by the AZD5363 alone, at the concentration of 0.3nM, as shown in Fig 5b. Furthermore, it is worth highlighting that at this concentration the drug alone had not reached the targeted IC_50_ value and the IC_50_ concentration of the complex is 10 times lower than that of the AZD5363 alone. Hence, the NS-AZD complex was identified as the most effective complex at a concentration of 0.3nM for further investigations. The selectivity of the drug carrier, i.e., NSCQD, when exposed at three different concentrations, i.e., 0.03, 0.3, and 3nM to healthy and cancerous cells was studied against Fibroblast and MCF7 cells, after 24 h. Although NSCQDs show biocompatibility at the tested concentrations for both healthy and cancerous cells, with a cell viability of above 87%, they behave specific against MCF7 cells, as shown in Fig 5c. The FTIR of the NS-AZD complex, unveils the characteristic peaks of both NSCQD and the AZD5363 drug, like the significant N-H, COO- and C-H vibrations of the AZD5363 at around 3189 cm^-1^, 3008 cm^-1^, and 2817 cm^-1^, respectively, as highlighted with purple, blue, and green in Fig 5d, which were not observed in the NSCQD’s FTIR spectrum (Fig 2c). Furthermore, noteworthy changes in the stretching C = C aromatic and C = O vibrations of the NSCQD, are observed as highlighted with yellow and red in Fig 5d, as the result of the conjugation with AZD5363, in terms of wavenumber shift and transmittance strength, becoming weaker and stronger, respectively. The explained variations in the FTIR spectra admits an efficient drug loading, with 97% efficiency. There are many known mechanisms involved in conjugation of various drugs to CQDs, including covalent, non-covalent interactions and encapsulation techniques. However, in case of the NS-AZD complex, covalent interaction between hydroxyl O-H groups in NSCQD and the R–N = C = O isocyanate functional group of AZD5363 and the non-covalent π-π stacking between aromatic C = C rings connect the components of the complex together [40].

### AKT inhibition assessment

**Fig 6:**
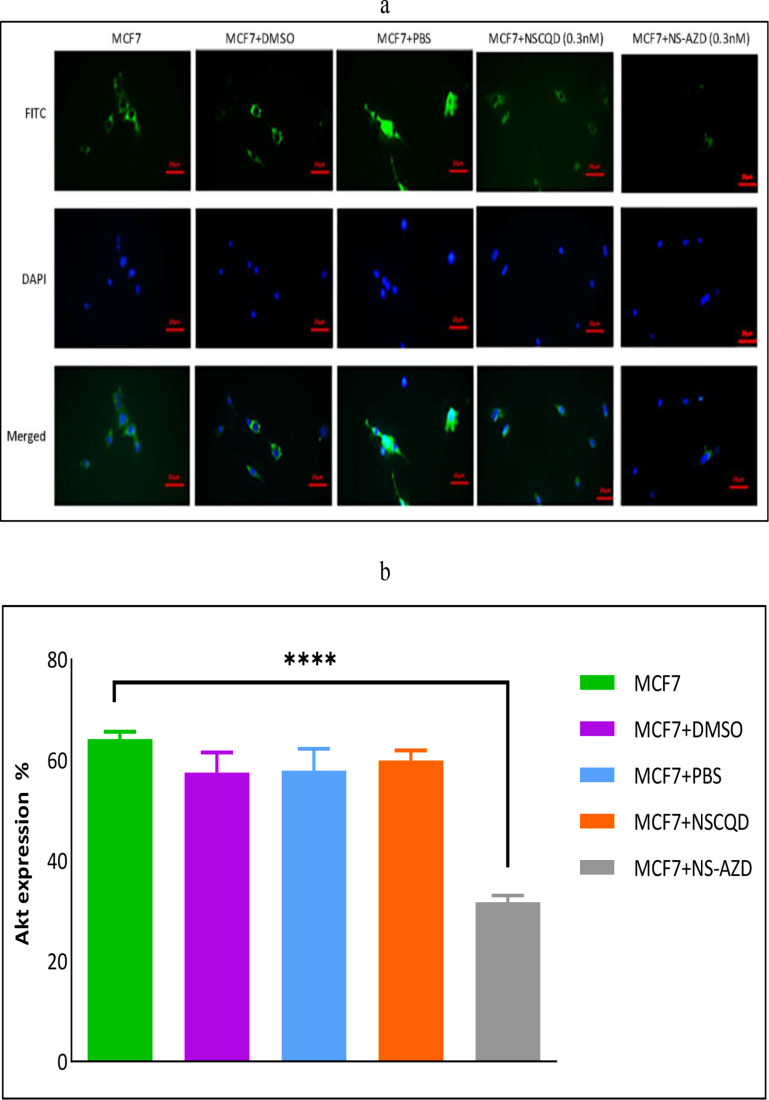
(a) Fluorescent microscopy of AKT expression at x100 zoom shown in green (first row); the cores shown in blue using DAPI filter (second row) and the merged mode presented in the third row; (b) Quantitative assessment of the AKT expression using DMSO-, PBS-, NSCQD- and NS-AZD (0.3nM)-treated groups (three replications).

The ICC assessment was used to evaluate the expression of AKT protein in MCF7 cells after treatment with DMSO (as the solvent of formazan), PBS (as the solvent of AZD5363 and NSCQD), NSCQDs (0.3nM), and the NS-AZD complex (0.3nM), as shown in Fig 6a. The results showed a statistically significant decrease (p-value < 0.0001) in the percentage of AKT protein expression in the MCF7 cells treated with NS-AZD (0.3nM) compared to the control group (Fig 6b).

### Apoptosis in MCF7 cells

**Fig 7:**
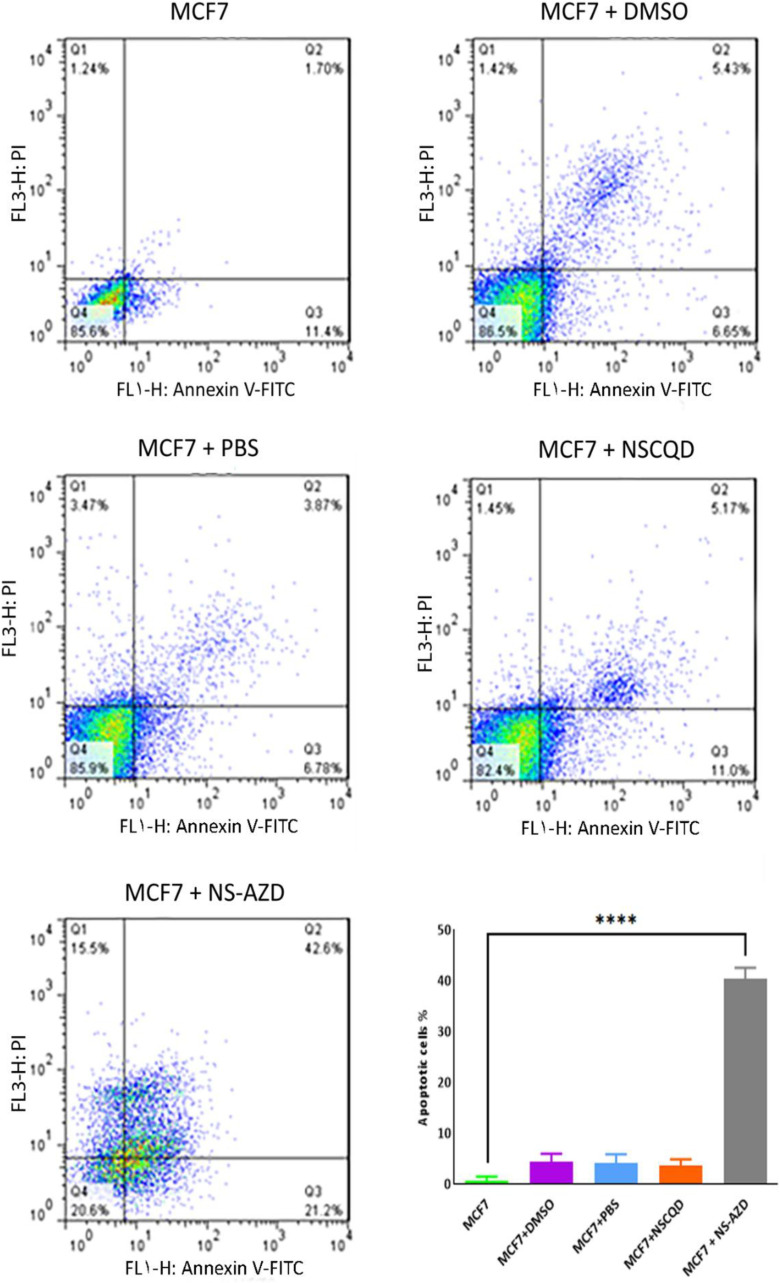
Flow cytometry results of induced apoptosis in the MCF7 cells alone and when treated by DMSO, PBS, NSCQD and NS-AZD (0.3nM), and the quantitative assessment of the induced apoptosis in the MCF7 cells after treatment with DMSO-, PBS-, NSCQD- and NS-AZD (0.3nM)-treated groups (three replications).

Flow cytometry was used to evaluate the rate of induced apoptosis in the MCF7 cell line in the selected groups. The results, as shown in Fig 7 indicated a statistically significant increase (p- p-value < 0.001) in the percentage of apoptotic cells in the NS-AZD (0.3nM) treated cells, compared to the control group. The percentage of apoptotic cells in other MCF7 cells treated with DMSO, PBS, and NSCQDs (0.3nM) were reported to be almost identical and no statistically significant difference was observed.

### Cell migratory capacity

**Fig 8:**
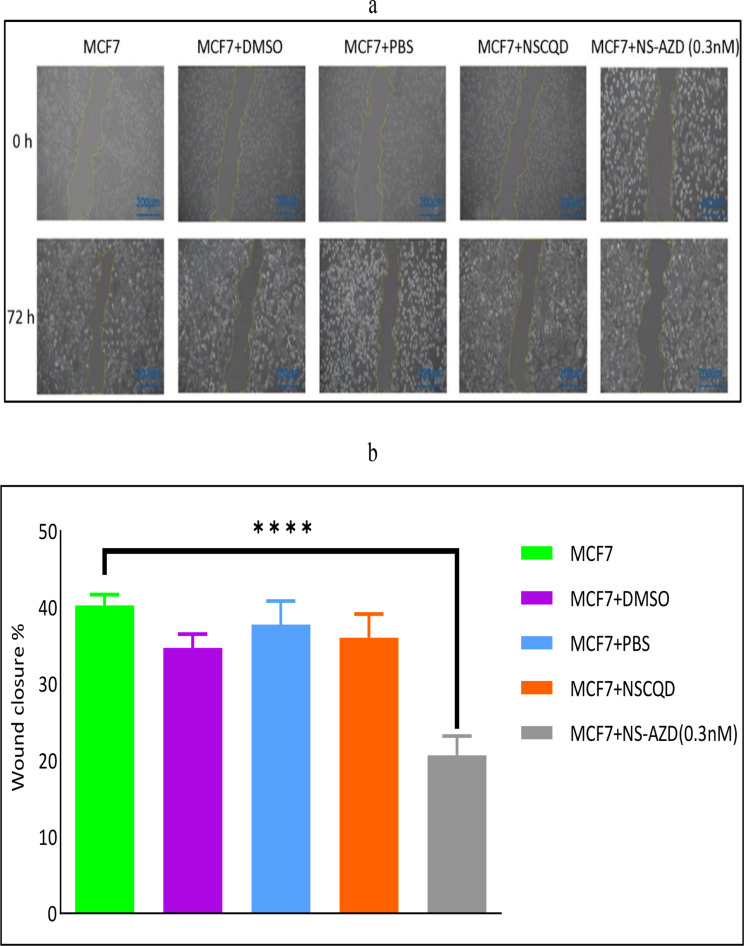
(a) The scratch-wound test (200μm scale-bar), and (b) the invasion rate results for the MCF7 cells, alone and when treated by DMSO, PBS, NSCQD and NS-AZD (0.3nM), initially and after 72 h. Standard deviation is calculated after three replications.

Finally, the cell scratch-wound technique was performed to study the impact of the NS-AZD complex on the metastasis of the MCF7 cell line. The invasion rates were measured initially and after 72 h for the targeted DMSO-, PBS-, NSCQD (0.3nM)-, and NS-AZD (0.3nM)-treated groups. The results showed a notable decrease in the progression of the scratched area in the NS-AZD (0.3nM)-treated group, in comparison with the control group, with a statistically significant difference (p-value < 0.0003) (Fig 8). The invasion rates in other treated groups, including DMSO-, PBS-, and NSCQD-treated MCF7 cells were almost identical to the control sample and no statistically significant difference was reported in the mentioned groups.

## Discussion

According to the findings of the last decade, surface properties and the final diameter of protein corona-decorated particles have a notable influence on the outcome of the tumour targeting strategy. There have been numerous studies demonstrating the low in-vitro and in-vivo cytotoxicity of CQDs, as a class of quantum-sized particles, introducing them as proper candidates for designing various drug delivery systems [41–44]. CQDs have good water solubility and can attach to various drugs through covalent or electrostatic bonds, utilizing their many surface-functional groups, which provide controlled release options and improved loading capability of drugs [45]. Furthermore, when using doped CQDs, owing to their diverse functional groups, nominal harm is induced in healthy tissues, providing specificity to cancerous cells, as confirmed in this investigation [11]. Hence, in this study, N-, S- and N/S-doped CQDs were synthesized using hydrothermal and pyrolysis techniques to investigate the influence of synthesis technique and doping on the properties of the newly developed drug carrier systems. Based on the results, N-, S- and N/S-doped CQDs were suitable in terms of size and expected variety in terms of functional groups. Using the synthesized CQDs, drug carrier composites were developed and studied to compare the effectiveness and selectivity of the newly developed systems.

Bases on the scientific concept of EPR, tumours often stimulate the formation of new blood vessels (angiogenesis) to supply the rapidly growing cancer cells, which are typically abnormal, with defective endothelial cells, wide fenestrations and a lack of smooth muscle layer, which allows larger molecules, up to about 200nm, to pass through the blood vessel walls more easily. On the other hand, tumour tissues usually have poor lymphatic drainage, which means that the larger molecules that have entered the tumour tissue, are retained there for longer periods, due to the incompletely developed lymphatic vessels, that are responsible for excreting them [46]. The size restrictions imposed by the EPR effect, sets the NCQD-AZD5363 composite out of the potential candidates, with average diameters of least 10% to 30% higher than 200 nm.

At this step, the rate of cell viability was studied using the MTT technique for the MCF7 cells treated with the synthesized CQDs, the drug alone and the AZD5363-CQD complexes at three different concentrations, i.e., 0.03, 0.3, and 3nM. Considering the IC_50_ concept, the NS-AZD complex is identified as the most effective complex at a concentration of 0.3nM. At this concentration, the NS-AZD complex provides a rather identical influence on cancerous cells viability, as the free AZD5363 does at ten times higher concentration. In other words, the complex shows an enhanced influence on the MCF7 cells, compared to the drug alone. Furthermore, in terms of selectivity of the drug carrier, the NSCQDs behaved more specific against MCF7 cells compared to healthy fibroblast cells. This contributes to the improvement of the therapeutic efficiency of the drug through an efficient drug loading and a controlled on-target release, due to an alternation in the pharmacokinetic factors of the drug, as admitted by FTIR results.

The FTIR of the NS-AZD complex unveils the mechanisms involved in conjugation of AZD5363 drug to NSCQDs. The covalent interaction between hydroxyl O-H groups in NSCQD and the R–N = C = O isocyanate functional group of AZD5363 and the non-covalent π-π stacking between aromatic C = C rings of the drug and the carrier connect the components of the complex together [40].

The enhanced influence of the proposed NS-AZD is also admits by the percentage of AKT protein expression in other treated groups, namely DMSO-, PBS-, and NSCQD-treated groups. The statistically significant reduction in the percentage of AKT protein expression in the MCF7 cells treated with NS-AZD (0.3nM) occurs due to reduced mTOR and p-Akt/mTOR expression by phosphorylation of 4EBP-1, as explained by Okuzumi et al. and Robertson et al., leading to a decrease in oestrogen receptor-mediated transcription as the primary chemotherapy strategy for breast cancer [20,47]. Enhanced phosphorylation of AKT was also demonstrated by the treatment of breast cancer cell lines with AZD5363 as a consequence of the protein being held in a hyperphosphorylated but catalytically inactive form following compound binding [12]. Hence, the strategy of incorporating NS-CQD with AZD5363 increases the AKT inhibitor’s activity of the drug and reduces its toxicity to healthy cells.

## Conclusion

This study highlights the promising potential of the NSCQDs as advanced drug delivery systems for breast cancer therapy. By combining the unique physicochemical properties of CQDs with the potent anticancer effects of AZD5363, a targeted AKT inhibitor, a system capable of efficient delivery of drugs to MCF7 cells was developed, with minimized harm to healthy cells. Compared to the drug alone, The NS-AZD complex demonstrated enhanced efficacy, as evidenced by a significantly lower IC50 (0.3 nM) confirming its superior therapeutic potential.

The biocompatibility of CQDs, supported by MTT assay results, underscores their suitability for medical applications. The high drug-loading efficiency of 97%, due to covalent interaction between hydroxyl O-H groups in NSCQD and the R–N = C = O isocyanate functional group of AZD5363 and the non-covalent π-π stacking between aromatic C = C rings of the drug and the carrier, further ensures minimal drug waste and efficient utilization, addressing common challenges in drug delivery systems.

Moreover, the size of the NS-AZD complex, measured at approximately 175 nm, falls within the optimal range for exploiting the enhanced permeability and retention effect. This characteristic would enable the complex to penetrate the leaky vasculature of tumors and remain in the tumor microenvironment for prolonged periods, facilitating sustained therapeutic effects. In addition, the NS-AZD complex exhibited the reduction of apoptosis induction, and inhibition of metastasis and cell invasion in MCF7 cells. These findings particularly highlight therapeutic value of this approach in estrogen receptor-positive (ER+) breast cancer.

In conclusion, the development of the NS-AZD drug delivery system represents a significant step forward in targeted breast cancer therapy. By addressing key challenges in specificity, efficacy, and biocompatibility. This study provides a strong foundation for the use of doped CQDs in advanced cancer treatment techniques. The findings presented here pave the way for future innovative, multimodal approaches that have the potential to transform the landscape of cancer therapy.
